# Better Understanding Insurance Mechanism in Dealing with Climate Change Risk, with Special Reference to China

**DOI:** 10.3390/ijerph18062996

**Published:** 2021-03-15

**Authors:** Feng Kong, Shao Sun

**Affiliations:** 1 College of Humanities and Development Studies, China Agricultural University, Beijing 100083, China; 2 Center for Crisis Management Research, Tsinghua University, Beijing 100084, China; 3 National Climate Center, China Meteorological Administration, Beijing 100081, China

**Keywords:** global climate change, coping with climate change risk, insurance mechanism, comprehensive disaster risk prevention, China

## Abstract

Climate change risk has become an important challenge for global sustainable development. The insurance industry can play an important role in coping with the increasingly severe climate change risk. This paper first describes the increasing climate change risk and the difficulties of the insurance mechanism in dealing with it. Then this paper summarizes the international practice of using the insurance mechanism to deal with climate change risk from ten different aspects. Based on the summary of the role of the insurance mechanism in dealing with this risk in developing countries, this paper puts forward the main application areas for climate change risk insurance and discusses the policy implications of developing climate change risk insurance in China.

## 1. Introduction

Global climate change has become one of the most serious challenges facing human society [1,2,3], causing frequent natural disasters such as temperature rise, sea level rise, hurricanes and rainstorm, which have caused great harm to public health, agricultural production, forest protection, water resources management and the ecosystem [4]. Global climate change not only causes great uncertainty, but also threatens the healthy global development of economy and society [5,6,7].

Since the second half of the 20th century, the number of global natural disasters has shown an obvious growth trend, and the economic losses caused by natural disasters have also shown a rapidly increasing trend [8,9], especially in economically underdeveloped areas such as East Asia, South Asia, Southeast Asia and Africa [10]. The global insurance coverage gap was $163 billion in 2018 [11]. The global insurance loss rate increased by 10% during 1979–2015, while total economic loss increased by 10.4% [12]. Total economic losses have grown faster than insurance losses over the past 35 years [3]. Therefore, in order to realize a strategy for natural disaster risk reduction and sustainable development, reduce the impact of natural disasters and establish a social system coexisting with risks, it is necessary to strengthen comprehensive disaster risk prevention. Disaster risk financing is an indispensable part of a comprehensive disaster risk prevention structure system. By raising a large number of funds, it can quickly provide these funds to the disaster area after the disaster occurs, so as to reduce the indirect impact of the disaster and ensure post disaster recovery and reconstruction, and effectively disperse the disaster risk suffered by individuals. In response to disaster risk, adequate financial preparation is the central link in a comprehensive disaster risk prevention system [13]. With the increasing frequency of natural disaster events and their increasing impact, the cost of disaster risk is increasing. The resource gap for post disaster relief and reconstruction is expanding [14]. The methods of risk reduction are not enough to effectively reduce the impact of natural disasters, especially catastrophes such as super large earthquakes, large-scale flooding and drought. Therefore, disaster risk financing is becoming more and more important in comprehensive disaster risk prevention [15,16].

The core idea of risk financing is to redistribute the disaster risk, to spread the disaster risk suffered by individuals or individual regions to other groups or regions, and usually to more people or larger regions [17]. The redistribution of disaster risk can be achieved through government actions, such as tax collection, a post disaster appropriation budget for reconstruction, etc., and can also be achieved through raising social funds [15]. Compared with the former, the use of social funds for disaster risk dispersion can reduce the opportunity cost as much as possible and reduce the dependence of disaster risk prevention and reduction on the government. The insurance mechanism is the first tool used by the international community to raise social funds for disaster risk dispersion. It is also the most important tool in developed countries. For example, the insurance mechanism is the most important way for developed countries to deal with moderate risks, including property insurance, crop insurance and national disaster insurance. Insurance companies will redistribute their risks in the form of reinsurance [16].

With the rapid development of financial markets in developed countries, disaster insurance started earlier in developing countries. Due to the spatial difference in global natural disaster risk distribution and the difference of the degree of financial market development in different regions of the world, the insurance mechanism plays different roles in disaster risk dispersion [16,17]. Generally, disaster insurance in developed countries can cover more than 30% of the losses after natural disasters, while in developing countries such as China the average disaster insurance coverage is only 1%. For example, the direct economic losses caused by Hurricane Katrina in 2005 and Hurricane Ike in 2008 in the USA were US $125 billion and US $38.3 billion, respectively, with insurance payments of US $62.2 billion and US $18.5 billion, respectively, accounting for 49.8% and 48.3% of direct economic losses. In contrast, the direct economic losses caused by the freezing snow rain disaster in southern China in 2008, the Wenchuan earthquake in 2008 and the Yushu earthquake in 2010 were RMB ¥151.7 billion yuan, ¥845.1 billion yuan and ¥64 billion, respectively, and the insurance compensation was RMB ¥5 billion, ¥1.66 billion and ¥0.8 million, respectively, accounting for 3.3%, 0.2% and 0.01% of the direct economic losses, respectively [10,16,17]. The average proportion of global insurance losses in total natural disaster losses was 43.6% during 2009–2014, i.e., 43.6% of natural disaster losses can be covered by insurance [17]. North America, Europe and Asia are the regions with the most serious natural disaster losses due to the long-term impact of earthquakes, hurricanes, floods, severe convective storms and other disasters. Among these, North America was the region with the highest loss and insurance compensation, and the proportion of loss covered by insurance compensation was very high, accounting for 62.6% on average during 2009–2014, the highest reaching 74.7%. Asia was the region with the second largest natural disaster loss, but the proportion of insurance compensation was very low, with an average of 11.2%, and the highest 18.9% in 2011, mainly due to the East Japan earthquake. Europe ranked third in disaster losses, with an average proportion of 35.6% and the highest at 49.8%. The average insurance compensation in Oceania was 50.6%, and the highest was 68.7% [17]. Generally, the insurance market in developed countries is proportionally well developed, and the proportion of natural disaster losses covered is much larger than that in developing countries [13].

At present, using insurance mechanism to disperse and transfer related natural disaster losses has become an important means for the international community to deal with climate change risk [2,18,19,20,21,22,23], while developing countries still lack the ability to use the insurance mechanism to realize the diversification and transfer of climate change risk [22].

**Figure 1:**
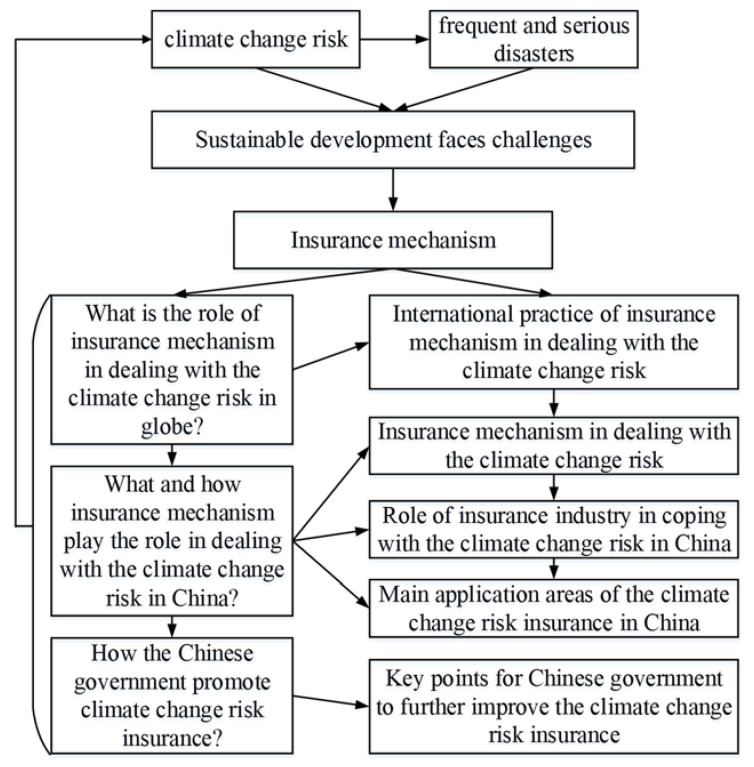
Insurance mechanism and climate change risk.

What is the core role of the insurance mechanism in dealing with climate change risk globally? What role and how can the insurance mechanism play in dealing with climate change risk in China? How does the Chinese government promote climate change risk insurance? These problems are not only the focus of current academic and practical circles [1,23], but also the core problems of this paper. Based on the above, this paper starts from international practice, from the aspects of international cooperation, climate change risk attribution, disaster risk model, disaster risk prevention, climate change risk insurance design, insurance products and services, carbon risk management and emission reduction services, climate protection, climate change risk financing and public policy-making to expound the application of the insurance mechanism in dealing with climate change risk. Then this paper briefly argues that the insurance mechanism can play a relevant role in climate change risk management, and takes China as an example to carry out a specific local summary. On this basis, this paper relates the insurance mechanism to climate change risk, explores the main application fields of climate change risk insurance in China, and puts forward important measures for the Chinese government to promote climate change risk (Figure 1).

## 2. International Practice of Insurance Mechanism in Dealing with Climate Change Risk

In the past 40 years, the number of natural catastrophes has increased significantly. In 1970, there were 100 catastrophes, but by 2017 the number of catastrophes had risen to 301. The insurance industry has a natural advantage in dealing with catastrophe. The international insurance industry has significantly strengthened its actions in response to climate change in recent years, mainly in the following 10 aspects.

**Figure 2:**
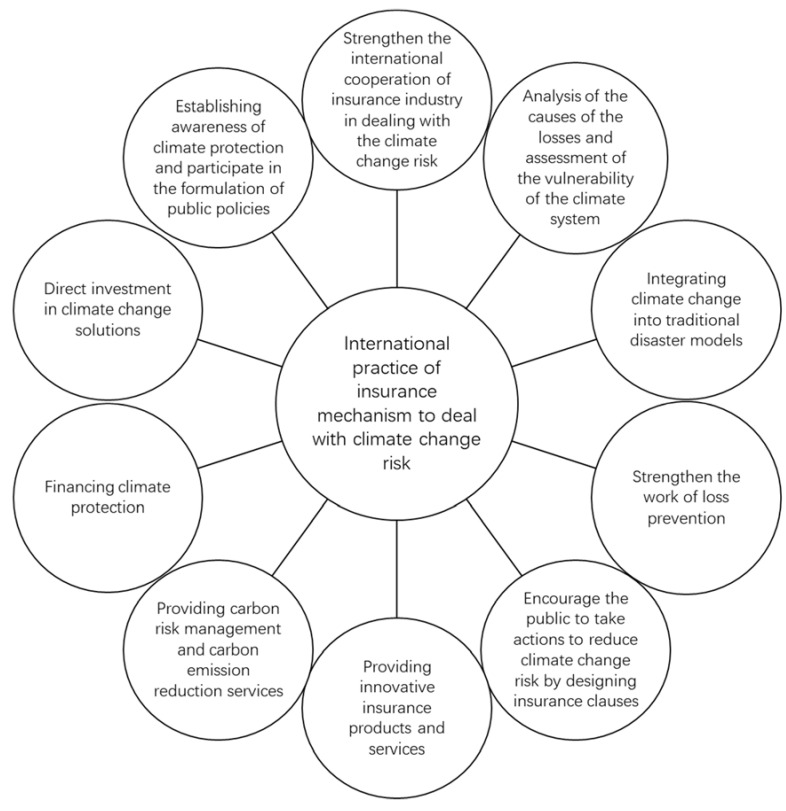
International practice of using insurance mechanism to deal with climate change risk from 10 aspects.

Firstly, the international cooperation of insurance industries in dealing with climate change risk has been strengthened. The establishment and development of early warning mechanisms for meteorological disasters in vulnerable areas has been supported, so as to deal with the disasters caused by climate change and reduce the economic and social risks (Figure 2) [24]. For example, some of the world’s major insurance companies set up the Climate Wise Cooperative Organization in 2006 to promote the insurance industry to better deal with climate change risk. The G7 Climate Risk Insurance Initiative is also proposed by the G7 group of seven developed countries, the United States, the United Kingdom, France, Germany, Japan, Italy and Canada. The goal of the initiative is to provide insurance for 400 million residents in developing countries to cope with the risk disaster crisis caused by climate change by 2020 [7].

Secondly, more and more insurance companies have begun to participate in scientific research on climate change risk, through field investigation and climate simulation model research, to explore the possible causes of disaster loss and assess the vulnerability of the climate system (Figure 2) [18,19]. For example, the Caitlin Group sponsored an Arctic expedition to measure the thickness and density of the Arctic ice sheet in 2009. Willis sponsored the National Center for Atmospheric Research of the United States to assess how global warming affects hurricane activity in the Gulf. The Ocean Holding Company of the University of Tokyo cooperated with the Climate System Research Center of the University of Tokyo to study the impact of global warming on typhoons using a climate simulation model [25].

Thirdly, the insurance industry has incorporated climate change into the traditional disaster model and carried out research on the physical effects and economics of climate change from the perspective of various hazards, disaster data, and a high-resolution climate disaster insurance model (Figure 2). Arkwright Mutual Insurance Company, as the first insurance company to study climate change, began to study flood data in the 1990s [12]. The Australian Insurance Company has cooperated with the University of Oklahoma to develop a high spatial and temporal resolution climate model. Munich Re has incorporated the physical effects of climate change into the hurricane model and studied the associated economic impacts, such as changes in demand and prices of building materials after disasters [26].

Fourthly, the insurance industry is increasingly involved in the process of adaptation to climate change risk, and is trying to play an important role in insurability in coastal and other high-risk areas (Figure 2). By supporting the development of green technology, it aims to mitigate the impact of sea-level rise and climate change on coastal communities, such as improving building codes and land-use management, and studying energy-saving and renewable energy technologies [5]. For example, American International Group has become a member of the New York City Panel on Climate Change, which aims to help cities develop strategies to address the impact of climate change. In 2008, the Heinz Center worked with Ceres to mitigate the impact of sea level rise and climate change on coastal communities [27]. In particular, land-use management means that insurance companies are participating in climate change risk prevention through government authorization, which includes managing land to increase carbon sequestration, thereby reducing greenhouse gas emissions and mitigating climate change itself, as well as managing land to act as, e.g., floodplains to mitigate the impacts of climate change. Insurance companies participate in land-use management through diversified channels and comprehensive measures [12,16,17]. Among these, comprehensive land use management measures, such as increasing grain yield, improving farmland management, improving grazing land management, improving animal husbandry management, improving forest management, increasing soil organic carbon content, managing fire, and reducing grain loss after harvest can prevent climate change risks and obtain higher benefits, with less side effects on land-use [16].

Fifthly, the insurance industry encourages the public to take actions to reduce climate change risk by designing insurance clauses (Figure 2). For example, the automobile insurance product based on mileage named Pay-As-You-Drive (PAYD) and its technology are very popular in Canada, Italy and the United States. PAYD can reduce mileage by 10–15% and reduce the traffic accident rate [28]. Regulators are also promoting the concept of PAYD. The California insurance regulatory agency has provided an optional “automatic payment” automobile insurance product for all consumers [29]. In Massachusetts, all insurance companies are required to offer a 10% discount on cars with an annual mileage of 5000 miles or less [29]. In addition, the American Association of Insurance Commissioners encourages insurers to give greater weight to policyholders’ driving miles in product pricing [22].

Sixthly, the insurance industry continues to provide innovative insurance products and services (Figure 2). In developed countries, the use of weather index insurance and weather derivatives for agricultural risk transfer is a new insurance product developed in recent years [29]. For example, the Security Investment Fund (SIF) of Alabama is the first catastrophe parameter insurance scheme signed by the government of an industrialized country. SIF can receive compensation from Swiss Re as long as the area is hit by a hurricane of category III or above. With the support of the World Bank and the Food and Agriculture Organization of the United Nations and other international organizations, developing countries have also actively explored agricultural weather index insurance and weather derivatives in recent years, which provides a new safeguard for agricultural risk transfer [3].

Seventhly, the insurance industry provides carbon risk management and carbon emission reduction services, as well as political risk and trade credit insurance for carbon emission trading (Figure 2). The risks involved include government intervention risk, embargo risk, license cancellation risk, and war and political violence risk, to production, certification and transportation related to the carbon credit line [1,30,31]. For example, RNK Capital LLC and Swiss Re jointly implemented the first carbon market insurance product to manage risk in carbon credit trading. The product provides insurance for the registration of clean and green development mechanism projects and emission reduction risks of certification issued under the Kyoto Protocol.

Eighthly, the insurance industry has provided funds for climate protection through diversified measures (Figure 2) [31]. For example, AXA MPS Assicurazioni Vita provides comprehensive risk coverage insurance products for photovoltaic systems involving loans, which mainly guarantees natural events caused by disasters, damage to photovoltaic systems, wildfires, and loss of income caused by lower output [3]. KBC provides preferential loans for owners to improve energy efficiency through its Green Energy Loan. Hong Kong and Shanghai Banking Corporation has provided funding for renewable energy projects, such as US $45 million for wind energy projects in India.

Ninthly, the insurance industry has begun to invest directly in solutions to climate change risk (Figure 2). For example, in 2007, Dresden Bank and the European Investment Bank launched the green bond project, which is expected to become the largest index linked joint bond in history, and the income from the bond was used to finance renewable energy and energy efficiency projects [23]. American International Group has invested in Sindicatum Carbon Ltd., a leading developer of greenhouse gas emission reduction projects. Dresdner Kleinwort Wasserstein first conducted carbon trading in the European market in 2004, and invested in trade and carbon compensation through the European Union carbon fund [32].

Tenthly, the global insurance industry and its regulatory agencies, through various actions, encourage the public to establish awareness of climate protection, so as to reduce energy consumption and greenhouse gas emissions, and participate in the formulation of public policies (Figure 2) [21]. For example, the American Association of Insurance Supervisors and Advocate of Highway and Vehicle Safety support telecommuting and increasing public transport funding to reduce energy consumption and greenhouse gas emissions. Insurance companies in Massachusetts offer extra benefits to consumers who use public transport [33]. If consumers buy a bus card for 11 months and use their personal car for no more than 10 days per month, they can enjoy a 10% premium discount. In addition, insurance policies will also have a great impact on the implementation of climate change policy. For example, renewable energy related insurance products implemented in the United States can enable more companies and investors to participate in renewable energy investment projects and fast-growing carbon emission trading projects [16,19].

In order to suppress and disperse climate change risk, the international insurance industry has developed a series of green financial products [34]. The green financial project itself is faced with high technical, market and policy risks. Green insurance is its bottom line of defense, which can be used to control the inherent market price risk of green financial products [35]. At present, green insurance in the international market mainly includes environmental liability insurance, green property insurance, catastrophe insurance and carbon insurance. Environmental liability insurance is the earliest type of green insurance, which is based on the compensation liability of enterprises in the event of pollution accidents [36]. Green property insurance is mainly for energy saving, new energy vehicles, and green building insurance. For example, Aviva Life Insurance Company Limited offers a 10% premium discount to policy holders for hybrid and fuel-efficient vehicles. The insurance products provided by Swiss Re can manage the risk of carbon credit price fluctuation, and they cooperated with an Australian insurance company to develop carbon delivery insurance products according to the emission reduction purchase agreement [3].

To sum up, the international community, through the above practices, hopes that the insurance industry can better promote society’s understanding of climate change risk, conduct in-depth and forward-looking thinking, and create solutions to minimize the impact and risk of climate change.

## 3. Diversified Role of Insurance Mechanism in Climate Risk Management

The insurance mechanism can play an important role in climate risk management [37]. A study by the World Bank compared the trend in GDP changes and the growth patterns of post disaster economies in countries with different insurance penetration rates. The results show that the GDP of countries with high insurance penetration rate shows a positive growth trend after experiencing a weather related catastrophe, which is in sharp contrast with those with low insurance penetration rate. After a catastrophe, the GDP of countries with low insurance penetration tends to show a negative growth trend. In addition, if there are no other economic growth factors to compensate, countries with lower insurance penetration may experience a long-term GDP recession after a catastrophe [3].

In response to extreme weather events, the insurance mechanism can play an important role in disaster loss compensation and risk prevention [38]. When using the insurance mechanism to prevent and manage the disaster risk of extreme weather events, different disaster risk bearers have different demands for insurance products. At the global level, it can form a global co-insurance agency, establish a global risk fund pool, and deal with large-scale loss risk around the world. At the national level, the government can establish and improve the national catastrophe insurance system, establish corresponding national insurance funds, issue catastrophe bonds, etc. [10]. The insurance products suitable for larger communities, associations and companies are index insurance or weather derivatives. Regional insurance can solve the disaster risks caused by climate change in a specific area. For individuals and groups, it can carry out agricultural insurance, small insurance and personal insurance.

Especially for developing countries, according to the impact of climate risk on different industries and groups, as well as the long-term potential impact on economic and social development, we believe that the insurance industry can protect against climate change risk via agricultural insurance, weather index insurance, property and life insurance for urban residents, green insurance, micro insurance of inclusive finance, etc., utilizing insurance practice to provide disaster risk management solutions and related disaster risk insurance products as the carrier, in order to play a positive role in dealing with the climate change risk [15].

## 4. Role of the Insurance Industry in Coping with Climate Change Risk in China

**Figure 3:**
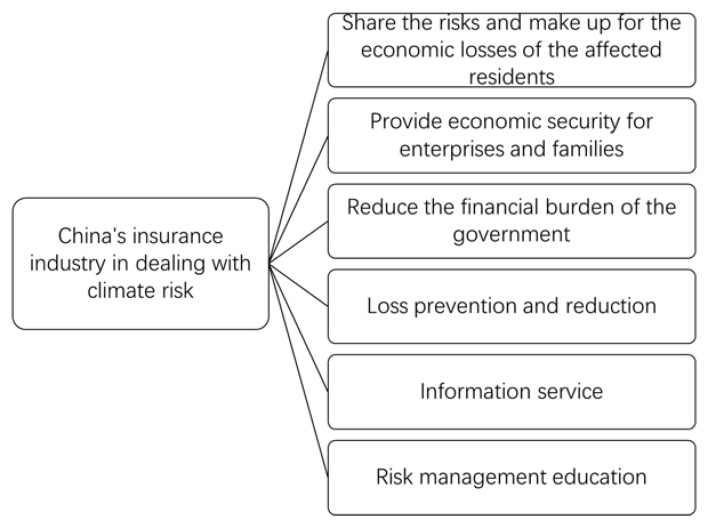
The role of China’s insurance industry in dealing with climate risk.

The insurance mechanism can disperse risks and make up for the economic losses of the affected residents (Figure 3). The most direct role of climate change risk insurance is to spread the disaster risk and reduce loss to the victims [39]. In recent years, with the increasing frequency and intensity of global climate disasters, coping with climate change risk has gradually become a common problem faced by the whole society. As a financial tool, climate change risk insurance has the function of dispersing risks, which can make up for the economic losses and personal injuries to the affected residents to a certain extent, and plays an important role in coping with climate disasters [1].

The insurance mechanism can provide economic security for enterprises and families (Figure 3). The main areas affected by climate change risk are economically underdeveloped areas, especially rural areas, which are more vulnerable to various risks and uncertainties. The insurance mechanism can provide financial security for farmers in economically underdeveloped areas, effectively reduce the impact of climate change risk, provide basic economic security for farmers, and provide an effective method of poverty alleviation [9].

The government’s fiscal stability can be guaranteed by its fiscal insurance mechanism (Figure 3). After the occurrence of climate disasters, disaster relief often has an impact on government finance, which may make the government fall into the dilemma of fiscal imbalance. Taking China as an example, at present China mainly adopts financial allocation and administrative guidance to carry out disaster prevention, mitigation and post disaster relief, which not only increases the financial burden at all levels, but also has low efficiency. As a market-oriented means, climate change risk insurance can make up for the deficiencies of the existing disaster prevention and relief system, help to improve the level of risk management and post disaster relief, and maintain the stability of the government budget [10,11].

The insurance mechanism has the functions of prevention and mitigation (Figure 3). The mechanism to deal with climate change risk is only one of link, and insurance can also play a positive role in disaster prevention and loss reduction. For example, the insurance companies will require the insured to reinforce a dam, improve building standards, carry out disaster prevention inspection activities, provide skills training in meteorological disaster prevention and meteorological knowledge, and help the insured improve their ability in disaster prevention and loss reduction. Climate change risk is uncertain, and the probability of extreme weather events is low, but the potential loss is huge. For these kinds of extreme weather event with low probability and high loss, it is a more cost-effective method to take pre-loss prevention and mitigation measures. In other words, we should make adequate preparation before the disaster, rather than relying on rescue afterwards, so as to reduce the possibility of disaster and the degree of loss as far as possible.

Insurance companies can provide information services (Figure 3). Information and data collection are the basis for assessing potential climate change risk. In addition, insurance companies can carry out meteorological disaster prevention services, provide meteorological information to insurance departments and policy holders, urge policy holders to take preventive measures against adverse weather, and reduce losses caused by meteorological disasters, so as to achieve the purpose of disaster prevention and reduction and reduce compensation.

The insurance mechanism can play a role in disaster risk management education (Figure 3) tthrough strengthening popularization and publicity regarding national climate change insurance knowledge, as well as strengthening communication and contact with the news media, actively carrying out publicity and education on climate change insurance, and improve residents’ awareness of climate change risk. For example, regular insurance knowledge training will be held to explain policies, terms, claims process and the relevant rights and obligations of the insured.

## 5. Main Application Areas of Climate Change Risk Insurance in China

According to the impact of climate change risk on different industries and different groups of people, as well as the long-term potential impact of climate change risk on global economic and social development, the insurance industry can carry out insurance practice against this background from the following points of view, so as to provide risk management solutions and related insurance products as the carrier, and give play to the positive role of the insurance industry in dealing with climate change risk.

**Figure 4:**
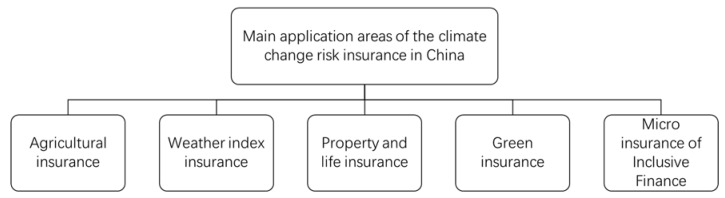
Main application areas of climate change risk insurance in China.

Firstly, climate change risk insurance can be applied in the field of agricultural insurance (Figure 4). Agriculture is the foundation of the national economy. Climate change has brought many adverse factors to agricultural development. For example, global warming will aggravate the shortage of agricultural water and restrict the rapid development of agriculture. North China is a region of water shortage. With the increase of temperature and evaporation, the water deficit will make the winter wheat region affected by water stress in North China expand southward, and scope of suitable areas for water supply will narrow, and the contradiction between supply of and demand for water resources will become more prominent [12]. Global warming will reduce the output of rice, wheat and corn. It is estimated that by 2030 China’s crop output may decrease by between 5–10% [15]. By 2071–2100, China’s agriculture will be even more impacted. The production potential of winter wheat will decrease by 10–30%, that of rice by 10–20%, and that of corn by 5–10%. Global warming will improve conditions for overwintering of eggs [16]. The boundary for insect eggs affecting major crops in China will move northward. The survival rate of pests will improve, the number of pests will increase dramatically, the occurrence and migration period of pests will be advanced and the harm period will be prolonged. After global warming, the degree of pest damage will increase by 10–20%, and the grain yield reduction due to pests will be further increased [17]. Therefore, the world, especially the developing countries, needs to strengthen the development of agricultural insurance to provide all-round guarantees for stabilizing agricultural production and promoting stable economic development. The insurance industry can explore multi-level agricultural risk protection, promote agricultural insurance product innovation, and help target poverty alleviation. First, the insurance industry should take the lead in setting up relevant insurance research projects on the impact of global warming on the optimal production areas for main crops in a specific region, directly transform these research results into insurance products, and cooperate with the government to make relevant policies to further promote the products. For agricultural pests, the potential impact of global warming on agricultural production can be effectively shared by setting up agricultural insurance products for different regions. Second, insurance companies can continue to develop and implement weather index insurance products, and actively make links with local characteristics and agricultural products to accompany agricultural poverty alleviation projects. For example, weather index and compensation conditions can be improved to make insurance products more flexible. Third, the insurance industry needs to innovate agricultural insurance products [30]. For example, according to the production and consumption of vegetables, the insurance companies should develop vegetable insurance in line with regional ecology, hedge climate change risk through the “futures & insurance” model, develop income insurance products such as corn and rice, and price index insurance products. In addition, with the rapid development of modern leisure agriculture and rural tourism, the insurance companies can consider the development and promotion of tourism insurance for leisure agriculture and rural tourism.

Secondly, the climate change risk insurance can also be applied to weather index insurance (Figure 4). Weather index insurance refers to one or several meteorological factors as trigger conditions. When the trigger conditions are met and farmers suffer more than a certain amount of loss and report to the insurance companies, the insurance companies will pay an insurance premium to the insured according to the crop yield and profit and loss, corresponding to the meteorological factor index. Standard, transparent and flexible weather index insurance products can avoid market failure without on-the-spot damage checking. Therefore, weather index insurance provides another feasible option for the transfer of climate change risk. To develop climate change risk insurance in the field of weather index insurance, the insurance industry can focus on weather index insurance products and weather derivatives, and carry out pilot and promotion work in industries, such as electrical power, energy, agriculture, tourism, etc., where weather index insurance products are applicable. Weather derivatives can not only hedge the risks caused by weather factors, but also share their own business risks with insurance companies. The government and insurance companies can speed up the establishment and improvement of weather derivatives, a new financial instrument. For example, they can provide power companies with temperature index futures products to avoid the risk of sales decline caused by a cold summer. In addition, the rapid development of the Internet and big data in recent years has also created a good information environment for the weather index insurance market.

Thirdly, climate change risk insurance can be applied to property and life insurance for urban residents (Figure 4). The fifth assessment report of the Intergovernmental Panel on Climate Change points out that the risks related to urban climate change are increasing, which have and will continue to affect the operation of the urban lifeline system, the quality of human settlements, the safety of life and property of residents, and ecological security. Floods, typhoons, droughts and other natural disasters as well as public emergencies directly threaten the safety of people’s lives and property, but also bring great challenges to urban risk management. In the field of property and life insurance for urban residents, the development of climate change risk insurance can take catastrophe insurance as the carrier, and the government as the main body of catastrophe insurance and the main investor. Taking China as an example, combined with the existing problems in the pilot process of catastrophe insurance in China, the insurance industry should develop various forms of catastrophe insurance product according to the characteristics of various regions, consider the threat of catastrophe risk to personal safety, promote catastrophe insurance products for property and personal safety, and integrate insurance into the social risk management system and urban public safety management mechanism, further improving the ability of cities and the whole of society to resist risk. In addition, the loss of fixed assets, machinery and equipment, which are included in the property of enterprises other than families, caused by catastrophe should also be considered under the scope of catastrophe insurance.

Fourthly, climate change risk insurance can be applied to green insurance (Figure 4). Due to large compensation amounts, narrow coverage and the immature management mode of green insurance, the risk of this kind of insurance is higher than that of other commercial insurance. Therefore, government support plays a key role in the development of green insurance. In addition, green insurance has a strong effect on public welfare, so its development is inseparable from the support of government. To develop climate change risk insurance in the field of green insurance, we need the cooperation of government departments and insurance companies to accelerate the innovation of green insurance products, and vigorously develop environmental liability insurance, green project loan guarantee insurance, energy saving insurance, carbon insurance, PAYD automobile insurance and other forms of green insurance. In terms of the development of environmental liability insurance, the government should specify the specific areas of compulsory insurance and arbitrary insurance based on the actual situation of enterprises, and redetermine the rate of environmental liability insurance according to the characteristics of local climate change risk. In the development of carbon insurance, the government can guide and support insurance institutions to develop carbon emission pledge loan guarantee insurance, carbon emission trading performance guarantee insurance, etc., and open insurance funds appropriately to enter the field of low-carbon technology research and development. In the development of energy-saving insurance, we can introduce the climate change risk insurance mechanism in the field of green buildings, such as improving energy efficiency to reduce energy consumption. In the field of low-carbon consumption, policy support is also needed, for example, in supporting the purchase of more energy-efficient housing and household appliances, and small replacement vehicles.

Fifthly, climate change risk insurance can be applied to the micro insurance of inclusive finance (Figure 4). Micro insurance provides a feasible way for low-income families to transfer risks. By paying a lower premium, micro insurance can protect against poor health, meteorological disasters and property losses. The government plays a key role in promoting the development of the micro insurance market. The government can work with the insurance industry to develop innovative disaster risk transfer solutions. This mechanism can help manage the rising cost of natural disasters and their impact on society, and alleviate contingent liabilities in the government budget. The government can also play an increasingly important role in meeting the needs and requirements of extremely poor groups in society. The government can improve the availability of financial services for the low-income population, establish a sound regulatory framework, reduce obstacles such as higher capital requirements, intermediary licensing standards, and strict regulatory requirements, and promote the further development of the micro insurance market. By enhancing the insurance awareness of low-income groups, the government can help establish a demand-oriented micro insurance market.

## 6. Key Points for Chinese Government to Further Improve Climate Change Risk Insurance

Firstly, the Chinese government should play a fundamental and leading role in promoting climate change risk insurance, incorporate climate change risk insurance into global and national strategies for adapting to climate change, and strengthen research on climate change risk insurance. From the strategic position of actively adapting to climate change, the overall objectives, main application fields, relevant systems and policies for developing climate risk insurance can be clarified, and relevant policies and regulatory systems established and improved to promote the development of climate risk insurance. In addition, the government also needs to strengthen the work of disaster prevention and reduction pre-disaster, and guide social capital including insurance funds to support disaster prevention and reduction projects through public-private partnership.

Secondly, the Chinese government should vigorously promote the application of climate change risk insurance in related fields. It is urgent to improve the catastrophe risk dispersion mechanism as soon as possible, ensure the sustainability of agricultural insurance operation, improve the financial subsidy system for agricultural insurance, and develop crop full cost insurance and income insurance. In addition, the insurance industry also needs to innovate forms of agricultural insurance related to climate change risk, balance the relationship between personalized supply and demand, develop and design multi trigger insurance policies, strengthen the support of scientific and technological innovation, standardize claim management, and actively encourage the insurance industry to carry out innovative practice in weather index insurance, so as to create a good market environment. For insurance companies, weather index insurance should be improved as soon as possible.

Thirdly, the Chinese government should study the establishment and improvement of the urban catastrophe risk insurance system. Against the background of global climate change, increasing risk also challenges governments at all levels to build resilient cities. The insurance mechanism is an important means for cities to deal with climate change risks. Governments at all levels should clarify the important position of insurance in the overall planning of building a resilient city, provide insurance solutions in various specific measures, and give full play to the role of the insurance mechanism in key areas and key projects.

Fourthly, the Chinese government should actively encourage the insurance industry to develop green insurance. For example, the government should vigorously support the development of environmental liability insurance, carbon insurance, green project loan guarantee insurance, energy saving insurance, PAYD automobile insurance and other green insurance. The insurance industry should actively carry out innovation in green insurance products and services. For example, the adoption of an agricultural production mode that is conducive to improving the environment can be regarded as the key influencing factor of rate determination in agricultural insurance. Insurance funds should pay more attention to green industry investment and support the development of green industry.

Fifthly, the Chinese government should vigorously promote the development of micro insurance for low-income groups and encourage insurance enterprises to actively engage in micro insurance business. Insurance companies should improve the understanding of the value of micro insurance, actively develop micro insurance, research and develop targeted insurance products, such as external injury insurance, property loss insurance, income loss insurance, etc., in order to provide basic economic compensation for low-income people suffering from climate risk loss.

## 7. Conclusions

Using the insurance mechanism to disperse and transfer disaster losses has become an important means for countries to deal with climate change risk. This paper describes the international practice of using the insurance mechanism to deal with climate change risk, and summarizes its typical characteristics. It includes strengthening the international cooperation of insurance industries, analyzing the causes of loss and assessing the vulnerability of climate change systems, incorporating climate change into the traditional disaster risk model, strengthening disaster loss prevention, encouraging the public to take actions to reduce climate risk by designing insurance clauses, providing innovative insurance products and services, providing carbon risk management and carbon emission reduction services, funding for climate protection, direct investment in climate change solutions, awareness of climate protection, and participation in the formulation of public policies. This paper argues that the insurance mechanism can play a diversified role in climate risk management. Taking China as an example, this paper summarizes the role that the insurance mechanism can play in terms of dispersing risks to make up for the economic losses to the affected residents, providing economic security for enterprises and families, reducing the financial burden of the government, ensuring the stability of government finance, loss prevention and mitigation, information services, risk management education, etc. This paper argues that future climate risk insurance can carry out relevant practice in agricultural insurance, weather index insurance, property and life insurance, green insurance, micro insurance of Inclusive Finance, etc. Finally, this paper puts forward suggestions that the Chinese government should play a fundamental and leading role in promoting climate change risk insurance. It is urgent to vigorously promote the application of climate change risk insurance in related fields, establish a catastrophe risk insurance system, and actively encourage and develop green insurance.

## Data Availability

No new data were created or analyzed in this study. Data sharing is not applicable to this article.
